# Remarks, Gleanings, and Extracts

**Published:** 1874-06

**Authors:** T. Curtis Smith

**Affiliations:** Middleport, Ohio


### REMARKS, GLEANINGS AND EXTRACTS

BY T. CURTIS SMITH, M.D., MIDDLEPORT, OHIO

*Insulation in the Treatment of Rheumatism and Other Diseases.* By P. M. WAGENHATS, M.D., Lancaster, Ohio.—In the causation of disease we have closely established the relation of *heat* to the fluxes of the bowels. When *ozone* is in abundance in the atmosphere, we have learned to expect influenza and affections of the mucous membrane of the respiratory tract. We trace typhoid fever to the infection of potable water by sewerage; and we have seen scarlet fever follow the milkman's cart, and with the pabulum of life sow the seeds of death.

Rheumatism, asthma and malarial fevers have an acknowledged dependence upon the atmosphere as to temperature, barometric pressure, and the presence of that tertium quid, vaguely called miasm. Electric tension has also its influence, which all, sick or well, recognize in their own persons. So, also, that material manifestation of force we know as *magnetism* has, in all probability, a powerful influence in the causation of disease. Concerning this we have very few observations recorded. The field of research has rapidly enlarged as to its production of currents, etc., but it has never been studied as the *cause* of disease.

Whilst innumerable observations are on record as to its powers as a therapeutic agent, I have no theory to offer as to the *modus operandi* of the treatment to be detailed in the subsequent part of this article, but offer it as the truthful record of what is at present a limited experience, in hope that observations on this subject may be multiplied, and the facts lying in this direction may be studied.

On December 25, 1872, I was attacked with rheumatism of the ankle and knee joints in one limb, then the other. I treated myself actively by alkalies, opiates, etc., in the ordinary manner recognized as of the most value in the disease. I was unable to leave my bed for three months, could not walk until April, 1872, and did not fully recover until the warm weather of June. On the 16th day of December, 1872, I was again assailed by my tormentor; treated myself as before, and "I thought myself happy" that I was able to be out of room in eight weeks, privileged to hobble around the streets of our city with the aid of a cane. Warm weather again restored me to health, and during last summer and this winter I attended to my professional duties. On February 16, 1874, while congratulating myself that I should escape my annual attack, I was suddenly seized in the night time with severe pains in both ankles. In the morning I failed, after an ardent effort, to leave my bed. Fever was intense, as also the swelling of ankle and knee joints. A sense of coldness of the lower extremities existed, which was even more distressing than the pain caused by the swelling of the joints. This condition continued until the morning of the 18th. From the 16th to 18th I was unable to sleep. On the morning of the 18th I *insulated* my bed, by causing the legs of the bedstead to be placed in four glass tumblers. In four hours thereafter the harrowing sense of coldness disappeared, yet the pain still continued. A sense of warmth and perspiration set in, and that night, at ten o'clock, I fell into a profound sleep, wakening on the morning of the 19th bathed in a profuse, warm perspiration, without the aid of diaphoretics or anodynes. I steadily improved, and in a few days was out of my room. On February 23, I left home for Cincinnati, where I remained a week, during all of which time I felt neither pain or soreness in the articulations. I returned to my home on Saturday, and found next morning the disease returned. I at once insulated my bed, and in eight days was able to attend to my professional duties.

George C——, age sixteen, during the autumn of 1873, had an attack of rheumatism, affecting nearly every joint in the body, and affecting the mitral valves of the heart. In the latter part of January, 1874, he was able to resume his labors. March 8th he had a relapse. I treated him in the usual way, without sensible improvement. I then concluded to try insulation, which I was slow to do in any case save my own, because it seemed whimsical. In six days after instituting insulation he left his bed, and was able to make a jouruey of eighteen miles, which he did without discomfort.

Mrs. ——, for eighteen years a sufferer from asthma, which occurred every month, without obvious connection with the menstrual function. During the cold months of the year she suffers almost continually. She had a severe attack in March, of this year, and in casting around for some *new* remedy, I concluded to insulate her bed. She was relieved in a short time, and since then, for more than a month, she has slept uninterruptedly upon the insulated bed, and has had no attack since.

I am aware that "one swallow does not make a summer;" and so small a number of observations does not establish the value of any form of treatment; yet, when I was so speedily relieved when I expected to follow the old course, I think there is value in it, and report these cases, promising another installment, for I have several under treatment.—*Chicago Med. Examiner.*—*Clinic.*

*On the Use of the Uterine Sound in the Diagnosis of Organic Dilatation of the Uterus Predisposed to Hemorrhage.*—By J. B. C. Gazzo, M.D., of La Fourche, La.—The causes of hemorrhage are very numerous, without attending to the inherent circumstances of pregnancy and labor, as well as the various traumatic lesions of which metrorrhagia is the necessary and inevitable result. Many women are, as we know, subject, during the whole period from puberty to the critical age, to hemorrhages, whose causes it is difficult to ascertain, and which are generally attributed to a certain dynamic, a local plethora, a molimen, or to repeated excitation of the uterus. Authors have admitted, in view of this, a large number of occasional causes, the influence of which upon hemorrhage is beyond a doubt, but in order that these occasional causes, these congestive excitements, should produce hemorrhage, we have most generally, and unless they have of themselves great power, therefore, to admit the aid of a special organic predisposition. It is to these organic predispositions, which appear to have partly escaped the notice of some of the professional men, that I wish for a moment to direct the attention of your readers on this subject.

I have observed that in all women subject to hemorrhage there is found an abnormal dilatation of the inner orifice of the uterine neck. This dilatation is detected in the living by the assistance of the uterine sound. In fact, instead of feeling a circular resistance, as in the case of hearty women, when we try to pass the inner opening, the sound enters with as much ease as through the external opening; this practical consideration appears of considerable importance in relation to the diagnosis and prognosis of metrorrhagia or of uterine hemorrhage. We can readily conceive, as is already proved by experience, that the flooding will continue or return so long as the internal opening of the neck of the uterus has not returned to its physiological dimensions. This uterine dilatation, therefore, appears to me to be ranked as one of the most powerful predisposing causes of hemorrhage, and this fact should be borne in mind, either in the diagnosis and prognosis, or as a proper indication in the treatment of uterine hemorrhage. Then let me here add that the general conclusion of the investigation was satisfactorily ascertained in two hundred cases, within the examination and diagnosis of uterine exploration of the womb, by the test of the uterine sound, without causing any uneasiness.—*Medical and Surgical Reporter.*

*Formation of True Bone in Penis.*—By Dr. J. von Lenhossek, Pest. (*Virchow's Archives*, vol. 60, April 1, 1874.)—The occurrence of *true ossification* in the penis has not been proved till now; the doctor's case is, therefore, very interesting, the more so, as not simple bony plates, but a larger piece of bone, besides a cartilaginous body, were found *post-mortem* in a corpse, of which he only learned that the individual died from typhus, at forty-two. Both the cartilaginous and the bony formations were situated in the cavernous bodies. There was a dorsal bone, with a channel for the dorsal vessels, and three ventral bones, with channel for the urethra. They originated from the middle fibrous septum of the cavernous bodies. Their color was yellowish. The microscopic examination revealed three different layers in the bony substance. The external layer consisted of connective tissue and some elastic fibres. The middle layer was composed of the same elements, containing spindle-shaped cells. The inner layer was characterized by the *Haversian canals,* about which were grouped, in concentric lines, the bony canals, connecting with the larger cavities of the bone. The cartilaginous body originated, likewise, from the middle septum, had a white color, and consisted of connective tissue and many elastic fibres. As the place of the erectile tissue was partly taken by the new formations, erections, of course, were very much interfered with. At last, the doctor states, that Professor von Siegmund observed bony bodies in penis, but only in life. In nearly all these cases the patients were, or had been syphilitic. The seat of these formations was also the cavernous bodies of the penis. The professor considered them as complete ossifications of the lymph vessels.—*Detroit Review of Medicine and Pharmacy.*

*Some Sources of Syphiltic Infection.*—In an able article on this subject, Dr. J. Nevins Hyde advances these propositions—

First, that "the ordinary menstrual secretion of a syphilitic female is capable of producing chancre and its consecutive symptoms under such circumstances as would insure contagion from a primary sore." With regard to the contagiousness of the blood of syphilitic patients there can be but little doubt after the oft-quoted experiments of Prof. Pellizari, and as the menstrual discharge is a true hemorrhage and not a secretion, it is reasonable to infer that it must possess this property in common with the circulating fluid. Several interesting cases are given in which this was the only method, apparently, by which infection could have taken place. By this means some of those hitherto inexplicable cases can be readily understood, in which, after confrontation, it has been impossible to discover any traces of disease in the female by which the male could have been contaminated.

Secondly, as Dr. Hyde shows, "the secretion of a uterus, which is the seat of secondary syphilitic deposits, are capable of prodcuing chancre and its consecutive symptoms under such circumstances as would ensure contagion from primary sores." In this connection the author points out the uncertainty which obscures our knowledge as to how far or in what form the uterus is the seat of secondary syphilitic manifestations.

Thirdly, a gonorrhœal discharge in a syphilitic subject is capable of producing chancre and its consecutive symptoms under such circumstances as would ensure contagion from primary sores.—*American Journal of Medical Sciences*, Jan., 1874.—*Nashville Medical Journal.*

*A Simple Plug for Rectal Hemorrhage.*—Two years ago, under somewhat urgent circumstances, Furneaux Jordan, F.R.C.S., devised a plug, which he has found ready, simple and efficient, in checking hemorrhage from the rectum, whether recurrent or secondary. The tip of the fore-finger thrusts the centre of a thin white pocket handkerchief well into the rectum; large marbles of compressed cotton wool (or rag) are then gently pushed into the pouch in the rectum, until it is amply distended, and of a balloon shape. Expulsive efforts, or moderate traction on the handkerchief, increase its hemostatic efficiency. The wool marbles may be moistened with the perchloride of iron, if it be deemed necessary. A plug saves the necessity of pulling down the rectal wall with a vulsellum forceps, and tying the bleeding point—not a pleasant alternative, especially without chloroform. In twenty-four or forty-eight hours the rectum converts the balloon into a cylinder, which is readily withdrawn at pleasure.—*Surgical Inquiries.*—*Nashville Journal of Medicine and Surgery.*

*Phosphoric Acid in the Urine in Cases of Disease of the Brain.*—With the view of determining whether the excretion of phosphoric acid by the kidneys is in any way influenced by diseases of the brain, Dr. E. Mendel has carried out a series of experiments, the results of which are as follows:

The relation between the phosphoric acid excreted and the remaining solid constituents of the urine varies considerably, ranging from 2·49 to 3·93 per cent., averaging, therefore, 3·233 per cent. A greater quantity of this substance is excreted by night than by day. In chronic diseases of the brain, the daily excretion of phosphoric acid is diminished, both absolutely and relatively, as compared with the other solid constituents. In cases of maniacal excitement, there is an increase (absolute and relative) of the phosphoric acid; an increase is likewise noticed during apoplectic and epileptic attacks, and also after the administration of chloral and bromide of potash.

If a diminution of the amount of this acid is observed in cases of chronic disease of the brain, this diminution will generally be found to be dependent upon the decreased muscular activity, consequent upon prolonged illness. In other cases, it is to be attributed to the general deterioration and wasting of the system, the result of imperfect assimilation.—*Archiv für Psychiatrie.*—*Boston Medical and Surgical Journal.*

*Double Spleen and Kidneys.*—In a *post-mortem* examination made by Surgeon-Major G. W. Jameson, in the city of Ghazeepore, the following abnormalities were found. In addition to one healthy, well-developed spleen, there was a second smaller one, connected with the abdominal vessels by separate communications of its own, and situated between the ordinary spleen and the liver. The smaller was of a roundish shape, and had a distinct hilus. Weight of first spleen, 9 oz., 1 dr., 6 gr.; weight of second spleen, 1 oz., 1 dr., 30 gr. There were also found four kidneys; two of these were well developed, healthy, and in the usual situation, while the second pair were small, intensely inflamed, and situated lower down than the other. The four kidneys had each their separate arterial and venous attachments and ureters. Weight of the two normal kidneys, 5 oz., 4 dr.; weight of the two smaller, 1 oz., 4 dr., 24 gr. The bladder was exceedingly small, walls much hypertrophied, and the mucous coat somewhat inflamed.—*Edinburgh Medical Journal*, April, 1874.

Sir Thomas Brown says: "Riolanus (1550) observeth that a man deaf from a bad conformation of the organs of the eare, pricking his eares too deepe, unawares pierced the tympane membrane and moved or broake the little bones, and afterwards came to heare: and, thereupon, proposeth the question whether such a practise might not bee attempted, which I confess I should be very warie to encourage; and I doubt few have attempted that course which he also proposeth agaynst the *tinnitus* and noise in the eares; that is to perforate the *mastoides*, and so afford a vent and passage unto the tumultuating spirits and winds."—*Boston Medical and Surgical Journal.*

*Death from Lancing of the Gum.*—In the *American Medical Journal* for April are given the particulars of the death of a child, fourteen months old, from hemorrhage occasioned by the lancing of the gum over the molar tooth. The blood oozed from the divided gum for three days, in spite of all efforts to suppress it. The child was well developed, and healthy from birth, and no previous suspicions had been entertained of the existence of a hemorrhagic diathesis.

